# Mitochondrial-Targeted Triphenylphosphonium–Hydroxycamptothecin Conjugate and Its Nano-Formulations for Breast Cancer Therapy: In Vitro and In Vivo Investigation

**DOI:** 10.3390/pharmaceutics15020388

**Published:** 2023-01-24

**Authors:** Kunfeng Zhang, Jingxin Fu, Xiaorui Liu, Yifei Guo, Meihua Han, Meifeng Liu, Xiangtao Wang

**Affiliations:** 1 School of Pharmacy, Henan University of Chinese Medicine, Zhengzhou 450046, China; 2 Institute of Medicinal Plant Development, Chinese Academy of Medical Sciences & Peking Union Medical College, Beijing 100193, China; 3 Key Laboratory of Functional Molecular Engineering of Guangdong Province, School of Chemistry and Chemical Engineering, South China University of Technology, Guangzhou 510640, China

**Keywords:** mitochondrial targeting, hydroxycamptothecin, triphenylphosphine, nanoparticles

## Abstract

Mitochondria are involved in various stages of cancer cell diffusion and metastasis. Therefore, targeting tumor mitochondria with antineoplastic medicines to cause mitochondria to initiate apoptosis may be an effective strategy for cancer therapy. Here, in order to enhance the anti-tumor efficacy of the antineoplastic agent hydroxycamptothecin (HCPT), the mitochondrial targeting ligand 4-(carboxybutyl) triphenylphosphine bromide (TPP) was attached to HCPT by an ester linkage. The resultant TPP-HCPT (TH) conjugate could self-assemble into nano-aggregates, with a mean particle size of 203.2 nm and a polydispersity index (PDI) value of 0.312. The TH conjugate could also co-assembly with mPEG_3000_-PLGA_5000_ into nanoparticles (TH-NPs), with a mean diameter of 86.41 nm, a PDI value of 0.256 and a zeta potential of −0.125 mV. In contrast to HCPT injections, TH aggregates displayed enhanced cellular uptake, mitochondria-targetability and cytotoxicity against 4T1 cells, while TH-NPs showed even better improvement than TH aggregates. In the in vivo study, TH aggregates displayed higher anti-tumor efficacy in 4T1 tumor-bearing mice than HCPT injections (tumor inhibition rate, 55.71% vs. 69.17%), and TH-NPs displayed more superior anti-tumor effects (tumor inhibition rate, 80.02%). In conclusion, our research demonstrated that the TPP-HCPT conjugate and its nano-formulations, including TH aggregates and TH-NPs, may be a promising mitochondria-targeting anticancer medicine for cancer therapy. As far as we know, this is the first report in which TPP and HCPT have been conjugated directly for this aim.

## 1. Introduction

Mitochondria are not only the main source of adenosine triphosphate (ATP) production in cells, but are also involved in many complicated regulatory processes leading to apoptosis [1,2]. The Warburg effect shows that the function of mitochondria is closely related to glycolysis and aerobic metabolism of cancer cells [3,4]. Redox and calcium homeostasis modulation, as well as transcriptional regulation, are all supported by mitochondria. Mitochondrial malfunction can cause a rise in reactive oxygen species (ROS) production, a decrease in membrane potential (ΔΨm), a change in membrane permeability, and the release of cytochrome C into the cytoplasm, which triggers apoptosis for tumor therapy [5,6,7,8].

The mitochondrial membrane acts as a vital barrier to the passage of medicines through the complex mitochondrial architecture [9]. Anti-cancer drugs can effectively enter mitochondria by means of the high mitochondrial membrane potential, protein input mechanisms, or mitochondrial targeting sequences (MTS), among which lipophilic cations are the most used and most effective way to deliver targeted medicines into mitochondria due to the high interior negative mitochondrial transmembrane potential of 130~150 mV. Many lipophilic cations accumulate preferentially in mitochondria; this then inhibits the transmission of the electron respiratory chain and initiates the apoptotic process. Compared to the mitochondrial membrane potential of normal cells (ΔΨm: −160 to −180 mV), the tumor cells usually exhibit a higher value (about −200 mV) [10]. Every 61.5 mV increase in membrane potential at 37 °C led to a 10-fold increase in the uptake of lipophilic cations via mitochondria [11,12], and this allows selectivity between normal cells and tumor cells.

The common lipophilic cations include triphenylphosphine (TPP), dequalinium chloride (DQA), rhodamine fluorescent dye, berberine and heptamethine cyanine dyes, among which TPP is the most frequently used [13,14,15,16,17]. TPP cations can easily pass through the mitochondrial membrane as the positive charges are dispersed over the large surface area of the TPP molecules, and potential gradients keep driving accumulation in the mitochondrial matrix [18,19,20]. Other reasons include their hydrophobicity and their low energy barrier for movements into the hydrophobic membrane core. 

Conjugation of TPP can effectively enhance both the accumulation of conjugated drugs in the mitochondria in vitro, and their cytotoxicity against many tumor cell lines. TPP modification on the surface of nanoparticles is another strategy for delivering cargo into cellular mitochondria and improving in vitro and in vivo antitumor efficacy [21,22]. However, the size of the nanoparticles may not be suitable for targeting intracellular mitochondria since the outer membrane of mitochondria can only support tiny molecules with a molecular weight of less than 5 kDa [23,24]. The conjugation of TPP with small molecular anticancer drugs is therefore still the mainstream method for targeting mitochondria without being affected by the outer membrane of mitochondria.

The antineoplastic medicine hydroxycamptothecin (HCPT), typically used to treat liver, stomach, and colon cancer, is an efficient Topoisomerase I inhibitor [25,26]. Topotecan and irinotecan are two HCPT-based antineoplastic targeted medications with some clinical success [27,28]. However, it is challenging for them to become clinical first-line therapies due to their limited water solubility, unstable chemical characteristics, and toxic side effects. It has been reported that HCPT can be formed into nanoparticles, micelles, liposomes, and other nano-dosage forms to improve the water solubility and stability of HCPT. However, these studies are still far from the therapeutic requirements [29,30,31,32].

**Figure 1:**
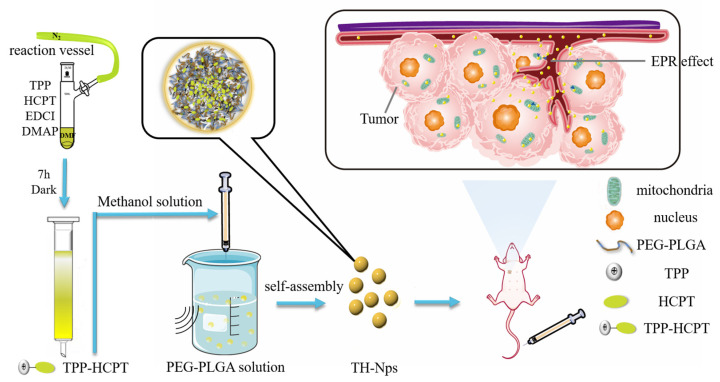
The schematic diagram showing the synthesis of TH, the preparation of TH-NPs by the anti-solvent precipitation method, how TH-NPs were in vivo delivered into tumor cells.

In this study, TPP and HCPT were coupled to enhance the tumor targeting of HCPT in cancer cell mitochondria (Figure 1). The resultant TPP-HCPT conjugate could self-assemble into nano-aggregates, which showed more improved anti-tumor therapeutic efficacy in vivo than free HCPT, but with limited stability. Further encapsulation of TH in a PLGA-mPEG copolymer using an antisolvent precipitation method created TH-NPs, which displayed excellent stability on shelf or in various physiological media, and further improved both in vitro anti-tumor activity and in vivo therapeutic efficacy.

## 2. Materials and Methods

### 2.1. Materials

10-Hydroxycamptothecin was purchased from Boer Chemical Reagent Co., Ltd. (Shanghai, China), and mPEG_3000_-PLGA_5000_ (PLGA:50/50) was obtained from Ruixi Biological Technology Co., Ltd. (Xi’an, China). HCPT injection was made according to commercial HCPT injection. (4-Carboxybutyl) triphenylphosphonium bromide, 1-Ethyl-3-(3-dimethylaminopropyl) carbodiimide hydrochloride (EDC. HCL), and 4-dimethylaminopyridine (DMAP) were purchased from Shanghai Aladdin Reagent Co., Ltd. (Shanghai, China). Coumarin 6 and the 3-(4, 5-dimethylthiazol-2-yl)-2, 5-diphenyltetrazolium bromide (MTT) were from Sigma-Aldrich (St. Louis, MO, USA). Mito-Tracker-Red was purchased from Beyotime Biotechnology Co., Ltd. (Shanghai, China). DIR iodide [1-1-dioctadecyl-3,3,3,3-tetramethlindotricarboc-yanine iodide] (DIR) was obtained from AAT BioQuest (Sunnyvale, CA, USA). All other chemicals were analytical grade.

### 2.2. Animals and Cell Lines

The 4T1 (breast cancer) cell line was obtained from the National Infrastructure of Cell Line Resource (Beijing, China). The cells were cultured in Roswell Park Memorial Institute (Buffalo, NY, USA) 1640 medium (RPMI 1640, Hy Clone) with 10% content of fetal bovine serum (FBS, Gibco, New York, NY, USA) with 5% CO_2_ at 37 °C. Female BALB/c mice (20 ± 2 g, 6–8 weeks old) were obtained from SPF (Beijing, China) Biotechnology Co. Ltd. (SPF grade).

### 2.3. Synthesis of TPP–HCPT (TH) Conjugate

**Figure 2:**
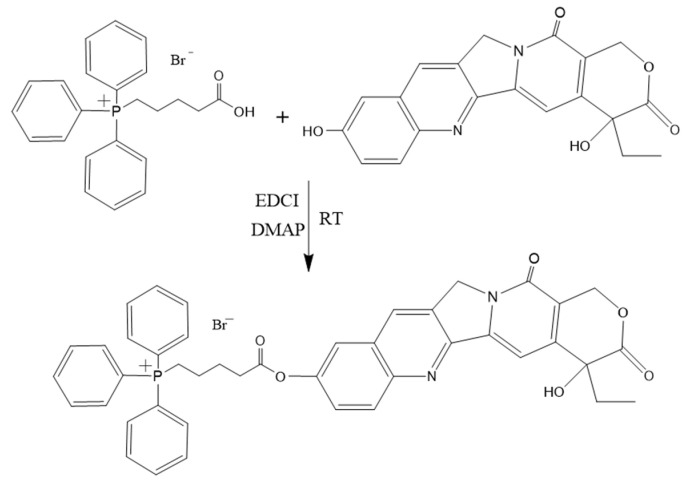
Synthesis scheme of TPP-HCPT conjugate.

HCPT (37 mg, 0.1 mmol) was completely dissolved in 1 mL of anhydrous DMF, and then EDCI (30 mg, 0.15 mmol), DMAP (2.4 mg, 0.02 mmol), TPP (67.5 mg, 0.15 mmol) were added under nitrogen protection and then reacted in the dark for 7 h (Figure 2). The progress of the reaction was monitored using thin-layer chromatography until the starting HCPT was almost completely consumed. Then, the reaction solution was concentrated, applied on a silica gel column (80 mesh silica gel, eluted by methanol: acetone (1:3), TLC examined at 365 nm) for purification. The structure of the goal product TPP-HCPT was determined using nuclear magnetic resonance (NMR) spectroscopy (Brucker, 600 MHz, Karlsruhe, Germany) and electron spray ionization mass spectrometry (ESI-MS, AXIMA-Assurance, Shimadzu, Kyoto, Japan).

### 2.4. HPLC Analysis

0.4 mg HCPT was dissolved in 100 μL DMF and diluted with anhydrous methanol to 4 mL, 0.4 mg TH was dissolved in 4 mL anhydrous methanol, and the samples were scanned at 190–700 nm wavelengths, respectively using a UV spectrophotometer (Cary 100 UV-VIS, Agilent Technologies, Santa Clara, CA, USA).

The HPLC system (DIONEX Ultimate 3000, Sunnyvale, CA, USA) was employed to measure the concentration of TPP-HCPT as well as HCPT. The Symmetry C18 column (250 mm × 4.6 mm, 5 µm, Venusil, SeparationTechnology Co. Ltd. Beijing, China) was used for chromatographic separation at 25 °C, and the mobile phase was 0.1% acetate water, methanol and acetonitrile (50:25:25 *v*/*v*) at a flow rate of 1 mL/min. The detection wavelength UV was 365 nm.

### 2.5. Self-Assembly of TPP-HCPT (TH) Conjugate

#### 2.5.1. Solubility and Nano-Aggregate of TH

TH was added into deionized water and sonicated for 5 min to examine its solubility and prepare different concentration of solution. The obtained TH solution was then placed in a colorimetric cell, and the particle size, polydispersion index, and zeta potential were measured by dynamic light scattering (DLS) using a Zetasizer Nano ZS system (Malvern Instruments Ltd., Malvern, UK). Each sample was measured in triplicate at 25 °C, and all data were expressed as mean ± standard deviation (SD).

#### 2.5.2. Critical Aggregation Concentration (CAC) Determination

The pyrene fluorescence method was employed to determine the CAC of the TPP-HCPT [33]. In short, 1998 μL of various concentrations (0.0025, 0.005, 0.01, 0.025, 0.05, 0.25, 0.05, 1, 2.5, 5, 10 μg/mL) of TPP-HCPT were combined with 2 μL of pyrene-acetone solution (0.1 mM). The mixture was then incubated at 37 °C for two hours to allow pyrene to reach the hydrophobic microzone. The fluorescence spectra of pyrene in the range of 350–500 nm were recorded using a fluorescence spectrophotometer (Techcomp, Shanghai, China), and the fluorescence intensities of pyrene at 373 nm (I1) and 384 nm (I3) were obtained. The hydrophobic microregion’s delicate change was characterized by the ratio of I1 to I3, and the CAC was the critical point before the value became stable. 

#### 2.5.3. HCPT Encapsulation by TH Conjugate

A total of 8 mg HCPT was dissolved into 0.8 mL mixed solvent (dichloromethane and methanol, volumetric ratio of 1:1), slowly dropped into 8 mL deionized water containing 8 mg TH under water bath sonication (250 W), followed by solvent removal under reduced pressure at 40 °C, and then homogenization at 1400 bar for 10 cycles.

For further hyaluronic acid (HA) coating, 2 mg of HA was dissolved in 0.2 mL deionized water, mixed with the above HCPT-loading TH nanoparticles (HA: HCPT = 1:4, mass ratio), stirred at 300 rpm for 10 min and then homogenized at 1400 bar for 5 cycles.

### 2.6. Preparation of TH Nanoparticles (TH-NPs)

#### 2.6.1. Improvement of TH Aggregate’s Stability by Co-assembly with Amphiphilic Polymer

We dissolved TH in methanol as the organic phase using the anti-solvent precipitation method and added various PEG block excipients as the aqueous phase. Next, we slowly dripped the organic phase into the aqueous phase under water bath sonication (250 W) at 37 °C. Methanol was removed using rotary evaporation under reduced pressure at 45 °C to obtain TH nanoparticles. We sought to obtain a nanoparticle with a particle size of 80–120 nm and PDI < 0.3.

#### 2.6.2. Preparation of TH Nanoparticles

TH can also co-assemble with mPEG_3000_-PLGA_5000_ into nanoparticles (TH-NPs) through antisolvent precipitation. In brief, 5 mg of TH was dissolved in 500 μL of methanol, then slowly dropped into 5 mL deionized water containing 20 mg of mPEG-PLGA under water bath sonication (250 W) at 37 °C. Methanol was removed using rotary evaporation under reduced pressure at 45 °C to obtain TH-NPs.

The same method was adopted to encapsulate DIR or Co-6 in HCPT injection, TH aggregates and TH-NPs just by co-dissolving DIR or Co-6 with organic phase in methanol and then being dropped into deionized water. The feeding mass ratio of TH to DIR or Co-6 was both 40:1.

#### 2.6.3. Determination of Particle Size, PDI and Zeta Potential

TH-NPs were placed in a colorimetric cell, and the particle size, polydispersion index, and zeta potential were measured by dynamic light scattering (DLS) using the Zetasizer Nano ZS system (Malvern Instruments Ltd., Malvern, UK). Each sample was measured in triplicate at 25 °C, and all data were expressed as mean ± standard deviation (SD).

#### 2.6.4. Measurement of Drug Loading Content and Encapsulation Efficiency

The lyophilized TH-NPs were weighed and well dissolved in methanol to determine the concentration of TH by HPLC analysis. The drug loading content (DLC) was calculated according to the following formula:

DLC (%) = C × V/W × 100%

(C: concentration of TH, V: volume of methanol solution of lyophilized TH-NPs powder, W: weight of the lyophilized powder of TH-NPs).

Next, 1 mL TH-NPs were centrifuged in an ultrafiltration centrifuge tube (MW = 5000) at 5000 rpm for 10 min. The filtrate separated into the lower centrifuge tube was analyzed using HPLC as the concentration of free TH (C_free_). 0.1 mL TH-NPs were well mixed with 9 times the amount of methanol, the mixture was injected into HPLC for analysis, and then the total concentration of TH in TH-NPs (C_total_) was calculated. The feeding amount of TH for 1 mL TH-NPs (W_feeding_) was calculated according to the total feeding amount of TH and the accurate volume of TH nanoparticles (constantly volumed in a volumetric bottle). The encapsulation efficiency was calculated according to the following formula:

Encapsulation efficiency (%) = (C_total_ − C_free_) × V/W_feeding_ × 100%

#### 2.6.5. Morphology of Nanoparticles

The morphology of TH aggregates and TH-NPs wase observed with a JEM-1400 electron microscope (JEOL, Tokyo, Japan). Negative staining by phosphotungstic acid was carried out to prepare the sample. A drop of sample solution was added to the 300-mesh copper sieve, dried at room temperature, then dyed with uranyl acetate for 90 s. Then, the morphology was observed under the accelerated voltage of 120 kV.

#### 2.6.6. Stability of TH Aggregates and TH-NPs

TH-NPs were kept at room temperature for 7 days, during which the particle size and PDI value were measured every day to examine their storage stability.

The stability of TH-NPs in physiological media was also checked. TH-nanoparticles were well mixed with the same volume of 1.8% NaCl, 10% glucose (Glu), 2 × PBS (pH 7.4), or 4 volumes of plasma, simulated gastric juice (1% pepsin in 1 mol/L diluted HCl) and simulated intestinal juice (1% trypsin in pH 6.8 PBS, 0.01 M), respectively. The mixture was then incubated at 37 °C for 12 h for the particle size and PDI value measurement at specific time intervals. Each sample was measured in triplicate, and all data were expressed as mean ± standard deviation (SD).

### 2.7. In Vitro Drug Release

A total of 1 mL of HCPT injection, TH aggregate or TH-NP was placed into a dialysis tubing (MWCO = 8000–14,000 Da) and dialyzed against 100 mL PBS (0.01 M, pH 7.2–7.4) at 37 °C under continuous stirring of 120 r/min. At the specific time intervals, 1 mL of dialysate was taken for HPLC analysis, and an equivalent volume of blank release medium was also added. The release medium was replenished every 24 h. The cumulative in vitro drug release of HCPT from HCPT injection, and TH from TH aggregates or TH-NPs was calculative and depicted. The aforementioned tests were carried out in triplicate.

### 2.8. In Vitro Cytotoxicity Assay

The in vitro cytotoxicity of free HCPT, free TH, TH aggregates and TH-NPs against the 4T1 cell line was performed with an MTT assay. In brief, 4T1 cells (3000 cells/well in 200 µL) in the logarithmic phase were seeded in 96-well plates and incubated at 37 °C in a 5% CO_2_ atmosphere for 24 h. RPMI-1640 medium serves as the negative control. Different concentrations (0.01–100 μg/mL) of free HCPT, free TH (dissolved in DMSO, diluted with RPMI-1640 medium), TH aggregates and TH-NPs (diluted in RPMI-1640 medium) were added, and the plates were incubated for 72 h. Then, 20 µL of MTT solution (5 mg/mL, dissolved in PBS and sterilized by filtration) was added to each well and incubated for another 4 h. After that, the medium was replaced by 150 mL DMSO to dissolve the formazan by pipetting up and down several times. The absorbance value of the supernatant in each well was measured at 570 nm using the ELISA plate reader. The cell inhibition rate was calculated as follows:

cell inhibition rate = (1 − OD_samples_/OD_control_) × 100

where OD_samples_ was obtained for the cells treated by the TH samples, and OD_control_ was obtained for the cells treated by the culture medium. The half maximal inhibitory concentration (IC_50_ value) was calculated by Graphpad 5.0 (GraphPad Software, Inc, San Diego, CA, USA).

### 2.9. In Vitro Cellular Uptake and Colocalization Analysis

The 4T1 cells in the logarithmic phase were inoculated into a 24-well plate (2 × 10^5^ cells per well) and incubated at 37 °C in 5% CO_2_ for 24 h. The Coumarin 6-labeled HCPT injection, TH aggregates and TH-NPs were diluted to a final HCPT or TH concentration of 10 µg/mL in a serum-free medium and added into the wells, mixed and co-incubated for 1 h, 3 h, and 6 h. Then, the culture medium was discarded, and Mito-Tracker Red (100 nM) and 4-amino-2-phenylindole (DAPI, 5 μg/mL) of 0.4 mL was added, in that order. Finally, the cells were fixed in PBS using 4% (*w*/*v*, 0.4 mL) paraformaldehyde. Cellular uptake was observed using a Fluorescent inverted microscope (OPLENIC Photoelectric Equipment Co., Ltd. (Hangzhou, China). Fluorescence semi-quantitative analysis and colocalization analysis was performed on image J software (version 1.5, National Institutes of Health, Bethesda, MD, USA).

### 2.10. In Vivo Antitumor Efficacy

The in vivo antitumor activity was evaluated using 4T1 tumor bearing mice model. After one week of adaptive feeding, 0.2 mL of 4T1 cell suspension (1 × 10^6^ 4T1 cells) was subcutaneously injected into the right armpit of Balb/c mice (about six weeks, 18–22 g). When the tumor volume reached 100 mm^3^, the mice were randomly divided into 4 groups (8 mice in each group), and, respectively intravenously injected with 0.2 mL normal saline (negative group), HCPT injection (5 mg/kg of HCPT, as a positive control group), TH aggregates (5 mg/kg of equivalent HCPT) and TH-NPs (5 mg/kg of equivalent HCPT) through the tail vein every two days, for a total of up to seven doses. Throughout the trial, behavior and physical condition were assessed and the tumor volume was measured every two days throughout the experiment. The body weight was also tracked as a sign of systemic toxicity in the mice. The tumor volume was calculated using the formula V = (L × W^2^)/2, where L and W represent the largest and smallest diameters, respectively. At the end of the experiment, the mice were sacrificed unless otherwise specified, and the tumors were dissected and weighed to calculate the tumor inhibition rate (IR) according to the following formulas: IR (%) = (1 − tumor weight of treated group/tumor weight of the control group) × 100%.

### 2.11. In Vivo Bio-Distribution of TH-NPs

In order to study whether drug-loaded nanoparticles can improve the distribution of drugs in mice, DIR-labeled HCPT injection and TH-NPs (HCPT 5 mg/kg, the mass ratio of DIR to HCPT was 1:40) were injected into 4T1 tumor-bearing mice via the tail vein (3 mice in each group, the tumor volume was approximately 1000 mm^3^). Whole-body fluorescence imaging was performed on a living imager (Caliper Life Sciences, Hopkinton, MA, USA) at 1, 2, 4, 6, 8, 12, 24, 36, and 48 h. Then, the tumor and the major organs such as heart, liver, spleen, lung, and kidney, were immediately dissected, imaged and semi-quantified by fluorescence 48 h later.

### 2.12. Statistical Analysis

Unless otherwise stated, GraphPad Prism software was used for evaluating the statistical difference for all the data obtained. In vitro and in vivo results were analyzed using the *t*-test and one-way analysis of variance. *p* < 0.05 was considered statistically significant. 

## 3. Results

### 3.1. Synthesis of TPP–HCPT Conjugate

The mitochondria-targeting TPP-HCPT was synthesized as outlined in Figure 2. The successful conjugation of TPP-HCPT (TH) was proved by NMR (Figure S1) and electrospray ionization mass spectrometry (ESI-MS) (Figure S2). In contrast to free HCPT, the HCPT moiety of TH displayed the absence of a 10-OH signal (10.37 ppm), slightly higher (∆0.16–0.23 ppm) chemical shift values for aromatic hydrogens close to 10-carbon, and the nearly unchanged chemical shift values of the hydrogen close to 20-OH, which supports the successful coupling of TPP at the position of 10-OH of HCPT.

The molecular weight of TH was also confirmed by electrospray ionization mass spectrometry (ESI-MS) spectra (Figure S2). In the [M + H]^+^ mode, the molecular ion peak of TH appeared at 709.33 m/z, which was consistent with the calculated m/z of [TPP-COO-HCPT]^+^. The total yield of TH synthesis was 63.5%, and the purity of TH was measured by HPLC to be 94.6%. 1H NMR (600 MHZ, DMSO-d6) δ: 8.68 (s, 1H), 8.21 (d, 1H, J = 9.1 Hz), 7.84–7.95 (m, 15H), 7.78 (d, 1H, J = 3.2 Hz), 7.58 (dd, 1H, J1 = 9.0 Hz, J2 = 2.2 Hz), 7.35 (s, 1H), 6.58(s, 1H), 5.44 (s, 2H), 5.31 (s, 2H), 3.71 (t, 2H), 2.78 (t, 2H), 1.90 (m, 2H), 1.85 (m, 2H), 1.70 (q, 2H), 0.88 (t, 3H). 

### 3.2. The HPLC Analysis

The full-wavelength UV-Vis spectra of free HCPT and the synthesized TH are shown in Figure S3. We chose 365 nm to be the detection wavelength for HPLC analysis of both HCPT and TH.

In the HPLC spectrum, the HCPT peak and TPP-HCPT(TH) peak were, respectively at 6.72 min and 11.57 min (Figure S3). HCPT displayed a linear regression equation of y = 1.3257x − 0.3871 with a line range of 0.1–200 μg/mL and an R^2^ of 0.9999, while TH showed a regression equation of y = 0.2997x + 0.4029 with a line range of 1–200 μg/mL and R^2^ being 0.9996.

### 3.3. Conclusions

#### 3.3.1. Self-Assembly of TPP-HCPT (TH) Conjugate

##### Solubility and Nano-Aggregation of TPP-HCPT

The solubility of HCPT in water was extremely low (3.4 μg/mL), although TH was quite soluble in water and its apparent solubility easily exceeded 10 mg/mL (Figure S4). Further investigation found that the TH solution was not a “real” solution, but rather aggregates formed by TH molecules. As seen in Table S1, the average particle size and the surface potential of TH aggregates increased with the TH concentration.

##### Critical Aggregation Concentration (CAC)

**Figure 3:**
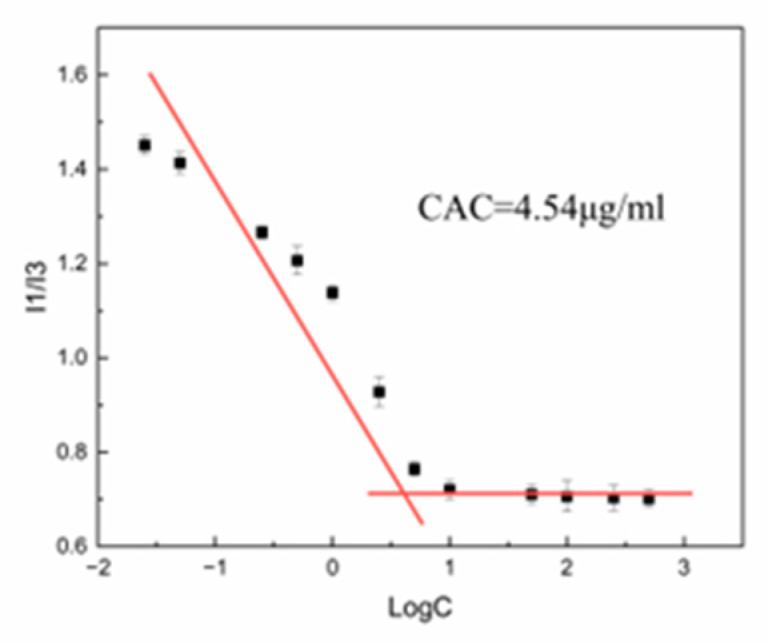
Determination of critical aggregation concentration (CAC) of TH using pyrene fluorescence method.

Pyrene is a suitable fluorescent probe for detecting the onset of micellization in micellar systems [34], it is hardly soluble in water (0.57 ± 0.03 μmol/L), and the amphiphilic substance can solubilize pyrene well in the resultant hydrophobic micellar core. At this time, the curve of the fluorescence intensity I1/I3 of pyrene will display a sudden change, and the concentration at this point was the CAC of the amphiphilic substance. Figure 3 depicts the relationship between the concentration of TH and the ratio of I1/I3. The CAC of TH was where the two lines connected; the value was 4.54 μg/mL (6.40 μM).

##### TH Aggregates

There was a quaternary phosphine salt cation in the chemical structure of TPP, and so the conjugation of TPP with hydrophobic HCPT resulted into an amphiphilic molecule of TH, which was able to form micelles-like aggregates. However, hydrophobic interaction may not be the main driving force for the formation of TH aggregates; the π-π stacking between HCPT moiety and the π-π stacking between TPP moiety may play a more important role. It has been reported that HCPT itself could form nanocrystals without the help of any pharmaceutical adjuvant, and so the average particle size could be well controlled to about 350 nm when finely operated [35]. Although TH aggregates could form nano-aggregates according to the same method (Figure S4), HPLC analysis found TH degradation during this process (data unshown), and so that method was considered unsuitable for preparing aggregates or TH nanocrystals.

**Figure 4:**
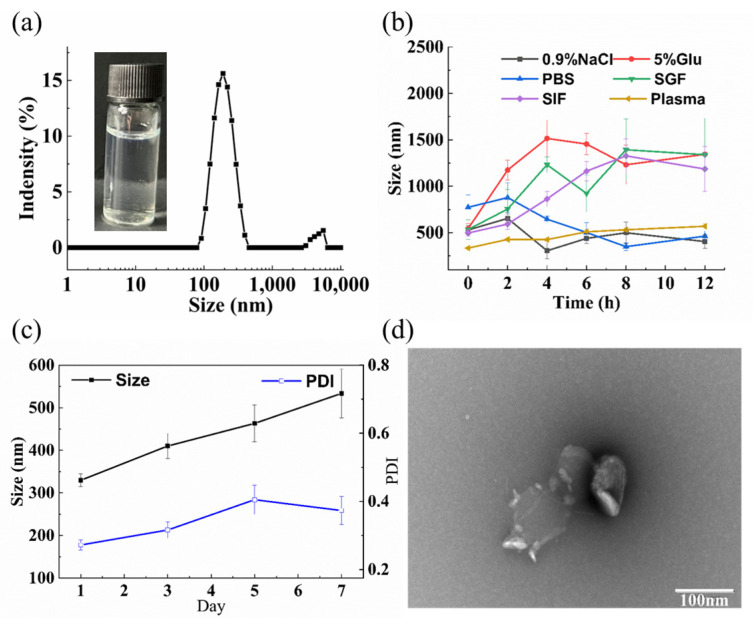
The actual photo and the particle size distribution of TH aggregates (**a**). The particle size change of TH aggregates in various physiological media (**b**). The particle size and PDI change of TH aggregates during the 7 days shelf storage (**c**). Transmission electron microscopy image of TH aggregates (**d**).

TH aggregates displayed a mean particle size of 203.2 nm, a PDI value of 0.272, and a zeta potential (+0.026 mV) in deionized water at 1 mg/mL of TH (Figure 4a). In 0.9% NaCl or PBS, the particle size of the TH aggregates was above 500 nm at the beginning, a little too large for intravenous administration, especially for anti-tumor treatment (usually < 300 nm), before decreasing to about 400 nm during the subsequent incubation at 37 °C (Figure 4b). In plasma, TH aggregates showed a stable particle size of about 400 nm throughout the 12 h of incubation at 37 °C, which demonstrated that TH nanoparticles could barely be used for intravenous injection. However, on the whole, TH nanoparticles was unstable, as their mean particle size easily increased to 1200–1600 nm within 6 h of incubation in 0.5% glucose, simulated gastric fluid, or simulated intestinal fluid (Figure 4b). Meanwhile, the on-shelf storage of TH aggregates found a continuous particle size increment (>200 nm) within 7 days, and slight precipitation on the 10th day (Figure 4c). Thus, further excipient screening, formulation and process optimization is required to improve their stability for effective drug delivery.

##### TH Conjugate as Carrier to Load HCPT

The amphilicity of TH and the possible π-π stacking between TH molecule and HCPT molecule remind us that the TH conjugate could be used as a drug carrier to encapsulate HCPT so as to form nanoparticles with high drug-loading content and mitochondria-targeting. With the help of an anti-solvent method combined with homogenization, we obtained HCPT-loading TH nanoparticles (the mass ratio of HCPT to TH being 1: 1) (Figure S5), with a mean particle size of 155.5 nm, a PDI value of 0, 312 and a surface potential of +5.62 mV. As the nanoparticles were not very stable during storage (particle seize increased to 220 nm overnight) and their positive charge was harmful to the in vivo long circulation, hyaluronic acid (HA) was used to coat HCPT-loading TH nanoparticles (HA:HCPT = 1:4, mass ratio). The resultant HA coated nanoparticles were about 190.8 nm in diameter, showed a narrow particle distribution (PDI being 0.208) and were slightly negatively charged (−3.71 mV) (Table S2). Unfortunately, the HA coated nanoparticles were only stable in plasma, their diameter increased to about 400 nm in 5% glucose (Figure S5). Even worse, precipitation was observed when incubated with other physiological media such as 0.9% NaCl, PBS and simulated gastric and intestinal fluid. More research is required to optimize HCPT-loading TH nanoparticles.

#### 3.3.2. Preparation of TH-NPs

TH aggregates were not very stable during storage, and their positive surface potential was adverse to long circulation when intravenously administrated, so amphiphilic block polymers with PEG segment were used to co-assemble with TH into nanoparticles. It was supposed that the long PEG chains could shield the positive charge and enhance the stability of TH nanoparticles. 

**Figure 5:**
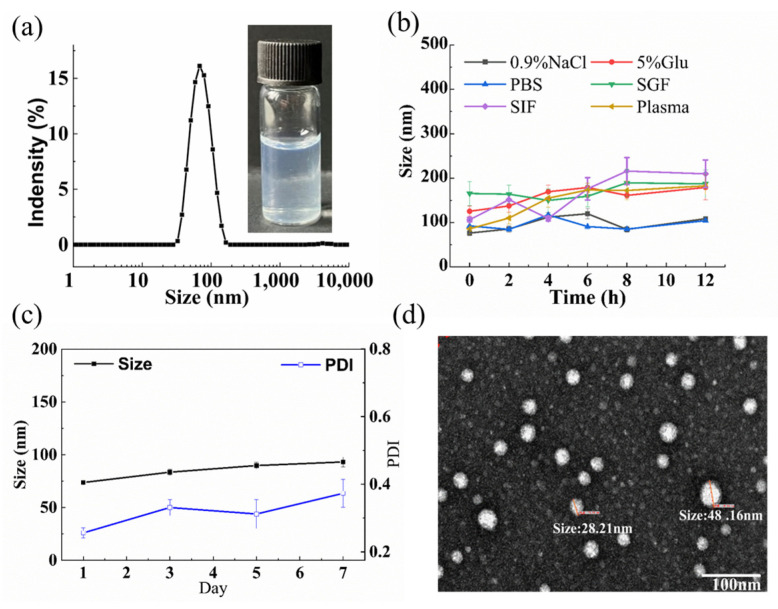
The actual photo and the particle size distribution of TH-NPs (**a**). The particle size and PDI change of TH-NPs in various physiological media (**b**). The particle size and PDI change of TH-NPs during the seven days shelf storage (**c**). Transmission electron microscopy image of TH-NPs (**d**).

As seen in Table S3, it was clear that the lower drug/polymer ratio led to smaller particle size and relatively lower PDI value. Among the seven kinds of pharmaceutical polymer, mPEG3000-PLGA5000 seemed to be most effective at preparing small and even TH nanoparticles. Unusually, further homogenization was not only unable to reduce the particle size of HA nanoparticles, but also increased particle size and worsen the size distribution. mPEG-PLGA shows good biocompatibility and biodegradability, and so was chosen to prepare TH nanoparticles in the subsequent experiment of this paper, with no homogenization being used. Considering that smaller nanoparticles usually penetrate deeper in the tumor, the TH/mPEG-PLGA mass ratio of 1:4 was adopted. The resultant TH nanoparticles (TH-NPs) had a mean particle size of 86.41 nm, a narrow size distribution (PDI value being 0.256) (Figure 5a), and a zeta potential of −0.125 mV. According to a study, nanoparticles with a suitable particle size (<200 nm) could help to avoid reticuloendothelial system uptake and achieve passive tumor targeting through the enhanced permeability and retention (EPR) effect [36,37].

The drug loading content of TH NPs was determined by HPLC to be 12.01%, and the encapsulation efficiency was 81.56%. TEM observation showed that the TH-NPs were well dispersed as individual nanoparticles with a regular spherical shape and the mean diameter was approximately 48.16 nm (Figure 5d), much smaller than that determined by DLS. This variation was attributed to the fact that TEM measurements gave the actual diameter of the dried particles while DLS measured the hydrodynamic diameter in aqueous phase. In comparison, TH aggregates showed irregular crystalline structure when observed by TEM (Figure 4d); this was probably due to the fact that TH aggregates, composed of merely TH molecules, were not very stable, and thus TH crystals formed during the sample preparation and the subsequent staining. 

By the help of mPEG-PLGA, the obtained TH-NPs were quite stable in all the six tested physiological media with insignificant particle size increment (Figure 5b). Furthermore TH-NPs displayed virtually no change in particle size during seven days storage at room temperature (Figure 5c). 

#### 3.3.3. In Vitro Drug Release Study

**Figure 6:**
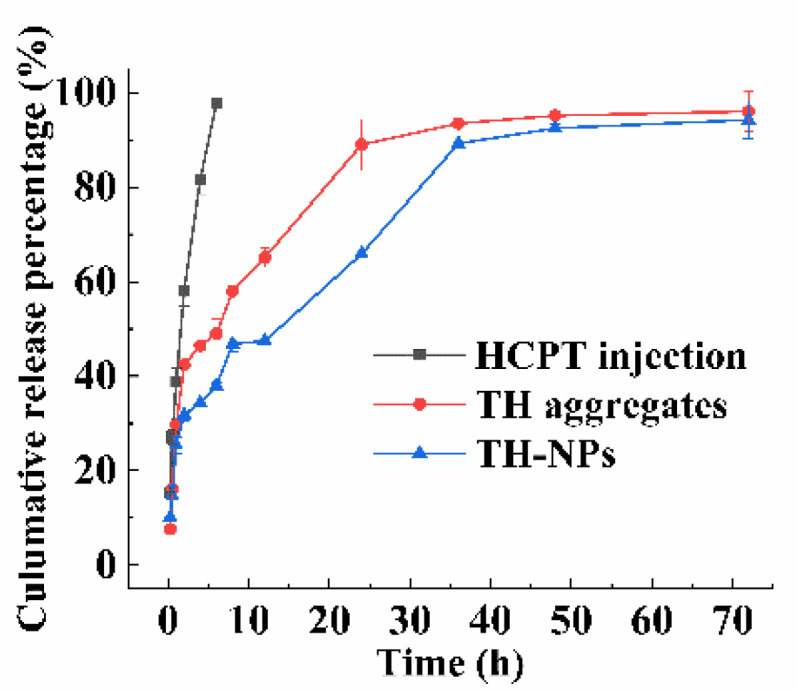
The cumulative in vitro drug release of HCPT injection, TH aggregates and TH-NPs in PBS (pH 7.4) (*n* = 3, mean ± SD).

Figure 6 illustrates the in vitro drug release of HCPT injection, TH aggregates and TH-NPs in PBS. Undoubtedly, HCPT injection showed a fast and continuous drug release. Both TH aggregates and TH-NPs displayed a two-phase drug release pattern, initially a burst release followed by a sustained drug release. In contrast to TH-NPs, HT aggregates had a bigger burst release (about 40% vs. 30%) and a faster release (65.10% vs. 47.45% within 12 h, 88.97% vs. 65.84% within 24 h). The burst release can be ascribed to the relatively higher hydrophilicity of TPP-HCPT (mainly for TH aggregates) and TH encapsulated in the outer layer in nanoparticles. 

#### 3.3.4. In Vitro Cytotoxicity Assay

**Figure 7:**
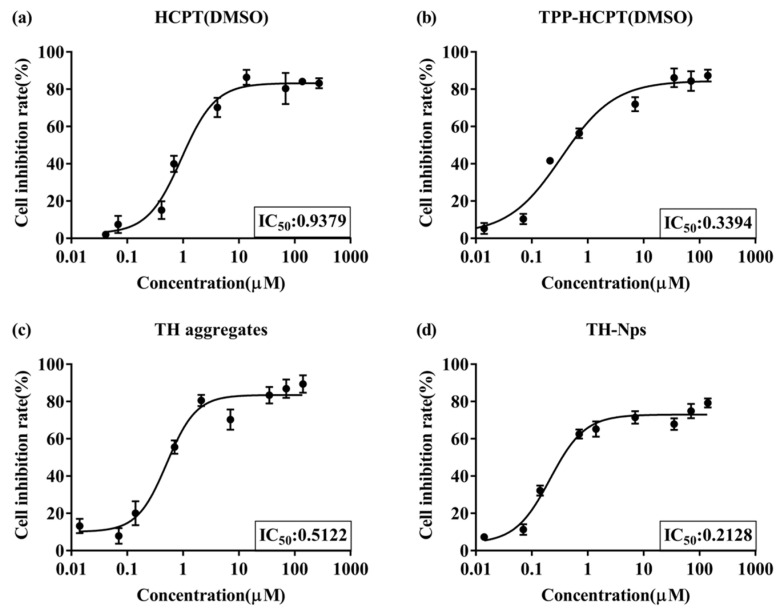
Cytotoxicity of HCPT (DMSO solution, (**a**)) and TPP-HCPT (DMSO solution, (**b**)), TH aggregates (**c**) and TH-NPs (**d**) against 4T1 cells after incubation for 48 h (*n* = 3).

An MTT assay (Figure 7) was performed to evaluate the cytotoxicity of free HCPT and free TH, TH aggregates and TH-NPs against a 4T1 breast cancer cell line (4T1 cell). TH showed a much higher inhibitory effect than free HCPT on 4T1 cells (IC_50_, 0.34 μM vs. 0.94 μM), likely due to the targeting effect of TPP on the mitochondria of tumor cells. In comparison with free TH, TH aggregates displayed a slightly higher IC50 of 0.51 μM while TH-NPs has a slightly lower IC50 of 0.21 μM.

#### 3.3.5. In Vitro Cellular Uptake Analysis and Colocalization Analysis

**Figure 8:**
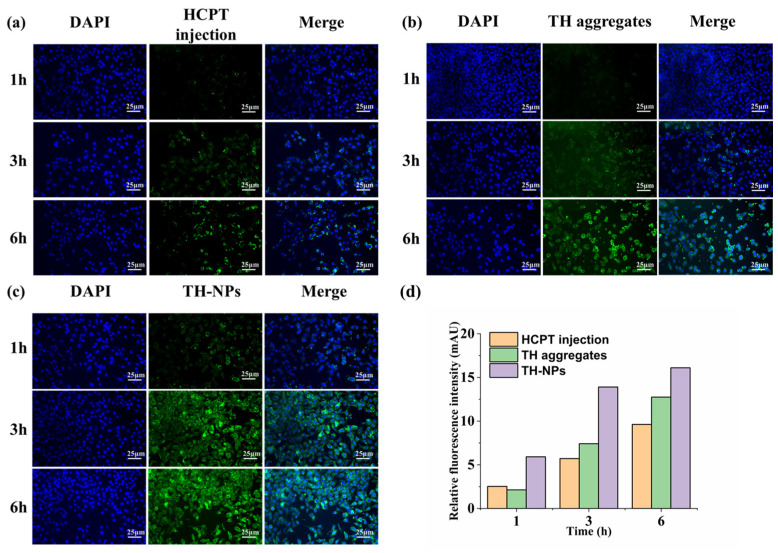
Fluorescent microscopy images for cellular uptake of HCPT injection (**a**), TH aggregates (**b**), TH-NPs (**c**) by 4T1 cells. blue: 40,6-diamidino-2-phenylindole (DAPI); green: Coumarin-6 labeled nanoparticles. The semi-quantitative analysis of cellular uptake of HCPT injection, TH aggregates and TH-NPs by 4T1 cells (**d**).

Coumarin-6 labeled HCPT injection, TH aggregates and TH-NPs were incubated with 4T1 cells for 1 h, 3 h and 6 h, respectively, to investigate their cellular uptake (Figure 8). Not surprisingly, for each dosage form, the 4T1 cellular uptake was time-dependent (Figure 8d). In contrast to TH aggregates, TH-NPs showed higher cellular uptake at all timepoints, which partially explained why TH-NPs had a lower IC_50_ than TH aggregates. 

**Figure 9:**
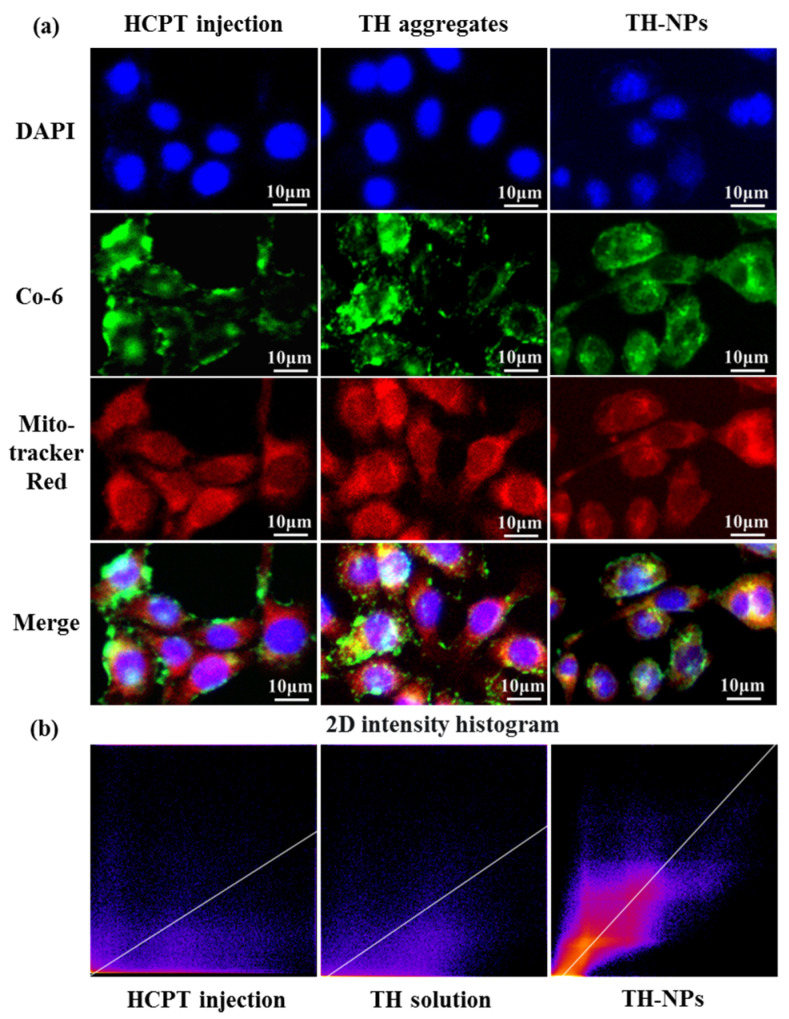
Co-localization analysis. The red fluorescence is the staining of the mitochondria in 4T1 cells, the green fluorescence is coumarin-labeled HCPT injection, TH aggregates and TH-NPs. The blue fluorescence is DAPI (**a**) 2D intensity histogram (orange points represent correlated part) (**b**).

An incubation time of 6 h was selected for mitochondria co-location analysis. As shown in Figure 9a, the yellow fluorescence of the overlapping images represents the co-localization of the Co-6-labeled experimental group and Mito-tracker Red. There was almost no overlap between the green and red channels in the HCPT injection group. TH aggregates had more yellow fluorescence, which shows the general mitochondrial targeting ability. On the other hand, TH-NPs have excellent selectivity to the mitochondria of 4T1 cells, and we can see that green and red fluorescence essentially coincide, which proves that it has superior mitochondrial targeting ability. 

The scatter plot is a commonly used methods in co-location analysis (Figure 9b). Therefore, we drew the scatter plot using the green and red channels with Fiji Image J software [38]. During HCPT injection, the scatter plot tends to diverge, and most of the points accumulate on the axis, demonstrating that the two variables are arbitrarily related in this situation. In contrast, when mitochondrial targeting occurs, the scatter plot shows a diagonal trend (y = x), representing a relatively strong positive correlation and a high degree of co-localization. Additionally, Pearson’s correlation coefficient (PCC) is also used as an index to quantify the degree of fluorescence co-localization. We calculated the PCC of green and red fluorescence in each group using Image J software. The HCPT injection group had a value of 0.13, the TH aggregates group had a value of 0.43, and the TH-NPs group had a value of 0.81. In general, we believe there is no connection between the two groups when PCC is 0–0.3, a low correlation exists when PCC is 0.3–0.5, and a high correlation exists when PCC is 0.5–0.8. The correlation between the two groups was quite strong (0.8–1.0).

#### 3.3.6. In Vivo Antitumor Efficacy

In this study, a 4T1 tumor bearing BALB/c mice model was used to explore the in vivo anti-tumor efficacy of TH aggregates and TH NPs, using HCPT injections as a control, all at the same dose of the equivalent HCPT. 

**Figure 10:**
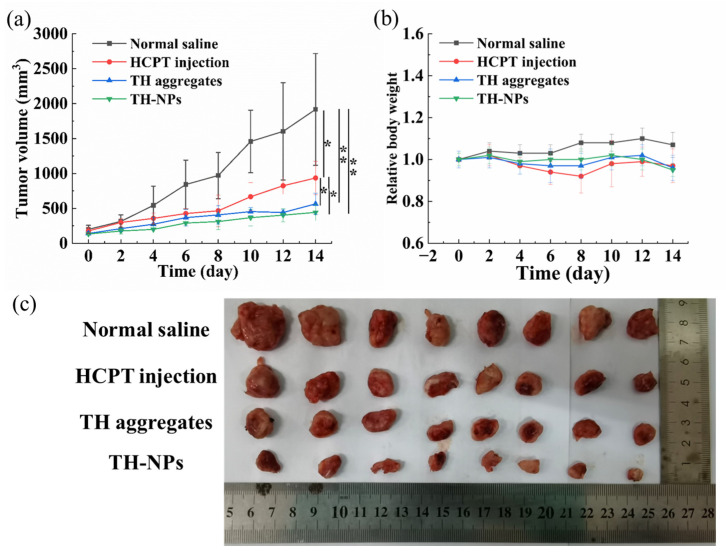
The tumor volume change, * *p* < 0.05 vs. normal saline. ** *p* < 0.01 vs. normal saline (**a**) and body weight change of the 4 groups of mice during the dosing period (**b**), and the actual photo of the tumors collected for the 4 groups at the end of the experiment (**c**). Data represent the mean ± SD (*n* = 8).

Figure 10a depicted the tumor volume change over 14 days of the experiment. The normal saline group showed very fast tumor growth, reaching 1917 mm^3^ within 14 days. However, mice treated with HCPT injections displayed much slower tumor growth, reaching only 935 mm^3^ at the end of experiment (*p* < 0.05). TH aggregates and TH-NP treatment led to even smaller tumor (about 564 mm^3^ and 443 mm^3^, respectively, both *p* < 0.01), displayed better anti-tumor efficacy than HCPT.

The tumors of the mice in each group were taken out at the end of the experiment (Figure 10c) and weighted for the calculation of tumor inhibition rate (TIR) on the basis of tumor weight. It was evident that the average tumor weight in each group followed the order of normal saline group >> HCPT group > TH aggregates group > TH-NPs group (Table 1). HCPT injection led to a tumor inhibition rate of 55.71% at a dose of 5 mg/kg. In comparison, TH aggregates resulted in a TIR of 68.50%, higher than that of the HCPT injection, demonstrating that conjugation with TPP could not only well dissolve the problem of poor solubility of HCPT, but also effectively improve the anti-tumor therapeutic efficacy of HCPT due to the mitochondria-targeting effect of TPP. Due to the smaller particle size and better stability, TH-NPs displayed even more improved anti-tumor efficacy with a TIR of 80.85% (*p* < 0.05), which was in line with the in vitro cytotoxicity result and cell uptake efficiency. TPP-modified HCPT may penetrate tumor cells more effectively and accumulate there. We speculate that mPEG-PLGA can not only stabilize the enormously rigid structure of TPP-HCPT, but also make it have longer blood circulation, higher tumor accumulation and higher cell uptake efficiency through the EPR effect. Overall, this is an anti-tumor strategy with great potential.

Figure 10b depicted the relative body weight change of mice over 14 days. Although there was no significant weight loss for each group, there was no obvious body weight increase either. In the whole, normal saline group displayed a very slight body weight increase and its average body weight was higher than those of all other groups at all timepoints due to the toxicity of HCPT [39,40], but no significant difference was observed. HCPT injection showed a relativly lower body weight. All the mice were in good condition during the treatment time, except for the HCPT injections group, in which about 60% mice showed frizz, curdling and less movement for a short time after dosing.

#### 3.3.7. In Vivo Biodistribution of TH-NPs

In order to study whether drug-loaded nanoparticles can improve the distribution of drugs in mice, a DIR-labeled HCPT injection and TH-NPs were injected into 4T1 tumor-bearing mice via the tail vein and then fluorescence imaged.

**Figure 11:**
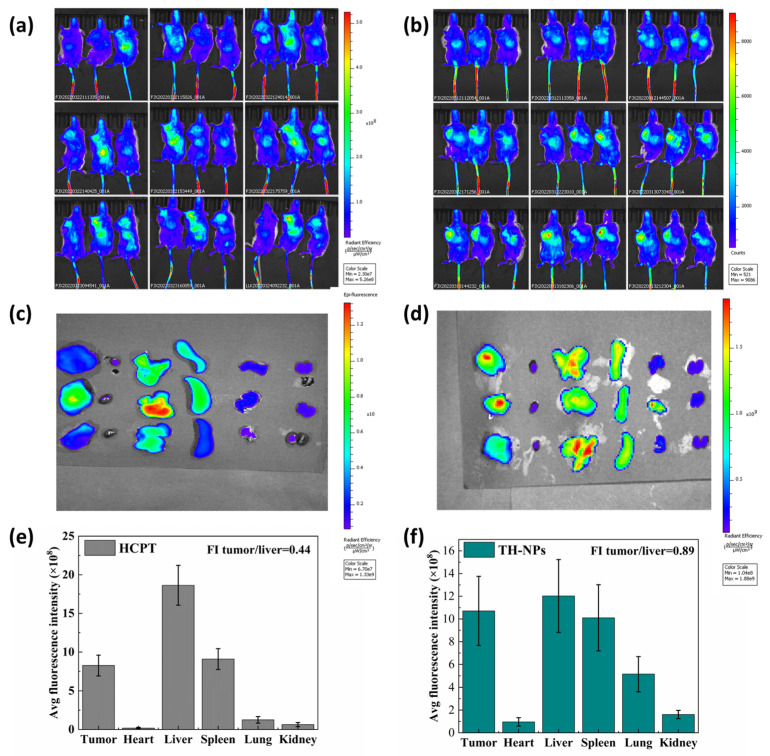
Dynamic biodistribution of DIR-labeled HCPT injection (**a**) and TH-NPs (**b**) in 4T1 tumor-bearing mice (from left to right: 1 h, 2 h, 4 h, 6 h, 8 h, 12 h, 24 h, 36 h, 48 h). The fluorescence image of dissected tumor and major organs (from left to right: tumor, heart, liver, spleen, lung and kidney) from HCPT injections group (**c**) and TH-NPs (**d**) at the end of the 48th hour, and the corresponding fluorescence semi-quantitative analysis ((**e**) for HCPT injection, (**f**) for TH-NPs).

As shown in Figure 11, in comparison to HCPT injections (Figure 11a), TH-NPs showed a significant accumulation of tumors from the 8th–24th hour (Figure 11b). This may be because of its smaller particle size and the long circulating effect provided by the PEG segment of mPEG-PLGA, which may stay longer in the blood circulation and so have more opportunity to accumulate in tumor. 

At the end of the experiment, the tumors and the major organs were dissected for direct imaging and more accurate data of the fluorescence intensity. As seen in Figure 11c,d, the liver showed the strongest fluorescence for the HCPT injection group, followed by spleen and tumor, while for TH-NPs, tumors displayed a fluorescence intensity close to the liver. The semi-quantitative fluorescence analysis using IVIS Living Image (Figure 11e,f) proved that the tumor/liver fluorescence intensity ratio of the nanoparticles group was 0.89, which was 2.02 times that of the control group (0.44), demonstrated that the TH-NPs had higher tumor targetability. This result is in line with the result of the in vivo anti-tumor efficacy study.

## 4. Conclusions

In this study, we combined the antineoplastic drug HCPT with the mitochondrial targeting ligand TPP to create TPP-HCPT nanoparticles, realizing the ability of mitochondrial targeting. This significantly improved the insolubility of HCPT and increased the drug’s ability to target tumor mitochondria. As far as we know, this is the first report that TPP and HCPT have been combined directly. Although TH can be assembled into nano-aggregates in water, the therapeutic impact in vivo was not optimum due to the instability and large particle size. The stabilizer mPEG-PLGA made up for these defects; the resultant nanoparticles helped TH benefit more from the EPR effect. The TH-NPs had an average particle size of 86.41 nm and a narrow particle size distribution, and were stable in various physiological media, and so were superior to TH aggregates. In vitro, TH was roughly 3-fold more toxic to 4T1 cells than HCPT, whereas TH-NPs were 4-fold more toxic. At the same time, TH-NPs had a higher uptake efficiency at 6 h, which was in line with the MTT results. Through the cell uptake experiment, it also proved that TH-NPs had superior mitochondrial targeting ability. The TIR of TH-NPs in the in vivo experiment was 80.85%, and even the TH aggregates had a tumor inhibition rate of 70.32%. Additionally, systemic toxicity was not observed in the whole animal experiment, except that the relative body weight change of the HCPT group was slightly larger. In the final vivo imaging system, the accumulation of TH-NPs in the tumor was double that of HCPT injection. After 48 h, the tumor’s fluorescence intensity was essentially the same as that of the liver tissue. These results suggested that the structural modification of HCPT by TPP can improve the selective cytotoxicity against tumor cells, mitochondria targeting and the in vivo anti-tumor efficacy. 

The synthesized TPP-HCPT conjugate, due to the presence of a quaternary phosphine salt cation in the TPP moiety and the excellent self-assembly property of the HCPT moiety, could easily form nano-aggregates. Additionally, TH aggregates can further encapsulate HCPT and its derivatives into carrier-free nanoparticles characteristic of mitochondria targeting. Further work is already underway to examine and optimize the encapsulation of more chemotherapeutics and/or immunomodulatory drugs and their combined anti-tumor treatment. Other valuable work is the tumor-targeting modification of the resultant nanoparticles to further improve the in vivo anti-tumor efficacy.

## Figures and Tables

**Table 1:** The antitumor effects against 4T1-bearing BALB/c mice; the results are presented as the mean ± SD, *n* = 8. * *p* < 0.05 vs. normal saline. ** *p* < 0.01 vs. normal saline. ^#^ *p* < 0.05 vs. HCPT solution.

Group	Tumor Volume (mm^3^)	Tumor Weight (g)	Inhibition Rate (%)
Saline	1917.5 ± 799.62	1.7015 ± 0.8492	—
HCPT injection	935.2 ± 240.7 *	0.7537 ± 0.3309 *	55.71 ± 15.92
TH aggregates	564.17 ± 153.1 **	0.5246 ± 0.1514 *	69.17 ± 8.90
TH-NPs	443.2 ± 118.0 **	0.3259 ± 0.1475 **	80.85 ± 7.49 ^#^

## Data Availability

Not applicable.

## Supplementary Materials

The following supporting information can be downloaded at: https://www.mdpi.com/article/10.3390/pharmaceutics15020388/s1, Figure S1: Proton NMR spectra of TPP(a), HCPT(b) and TPP-HCPT conjugate (c); Figure S2: ESI-MS of TPP-HCPT (ESI source: I Spary Voltage: 4.50 kV; Tube Lens: 110 V; Capillary Temp: 275 °C; Capillary Voltage: 35 V; Sheath Gas Flow Rate: 5arb); Figure S3: UV full-wavelength scanning of HCPT and TH (190–700 nm, a and b). High performance liquid chromatography (HPLC) of HCPT and TH under the exact same chromatographic conditions (c); Figure S4: HCPT and TH in deionized water (1 and 4), dissolved in alkaline solution (pH 8.5) (2 and 5), and the subsequent aggregation when acidified to pH 6.5 using 1M HCl; Figure S5: HCPT-loaded TH nanoparticles (a) and their particle size change in 5% glucose and plasma after HA coating (b); Table S1: Particle size and PDI of TH solution at different concentrations; Table S2: Particle size, PDI and ze’ta potential of HCPT-loaded TH nanoparticles and HA coated HCPT-loaded TH nanoparticles; Table S3: The particle size and PDI value of TH nanoparticles (containing 1 mg/mL of TH) made from different pharmaceutical adjuvants.
