# Supplementary material for: Mitochondrial-Targeted Triphenylphosphonium–Hydroxycamptothecin Conjugate and Its Nano-Formulations for Breast Cancer Therapy: In Vitro and In Vivo Investigation
Source: Pharmaceutics. 2023 Jan 24;15(2):388. doi: 10.3390/pharmaceutics15020388 (PMC9961676; doi:10.3390/pharmaceutics15020388)
Supplement: Supplementary file 1 [file pharmaceutics-15-00388-s001.zip › pharmaceutics-2141630-supplementary.pdf]

Supporting Information

Mitochondrial-Targeted Triphenylphosphonium–Hydroxycamptothecin Conjugate and Its Nano-formulations for Breast Cancer Therapy: in vitro and in vivo Investigation

Kunfeng Zhang[1], Jingxin Fu[2], Xiaorui Liu[2]， Yifei Guo[2], Meihua Han[2], Meifeng Liu[3,*] and Xiangtao Wang[1,2,*]

1 School of Pharmacy, Henan University of Chinese Medicine, Zhengzhou 450046, China.

2 Institute of Medicinal Plant Development, Chinese Academy of Medical Sciences & Peking Union Medical College, Beijing 100193, China.

3. Key Laboratory of Functional Molecular Engineering of Guangdong Province, School of Chemistry and Chemical Engineering, South China University of Technology, Guangzhou, 510640, China

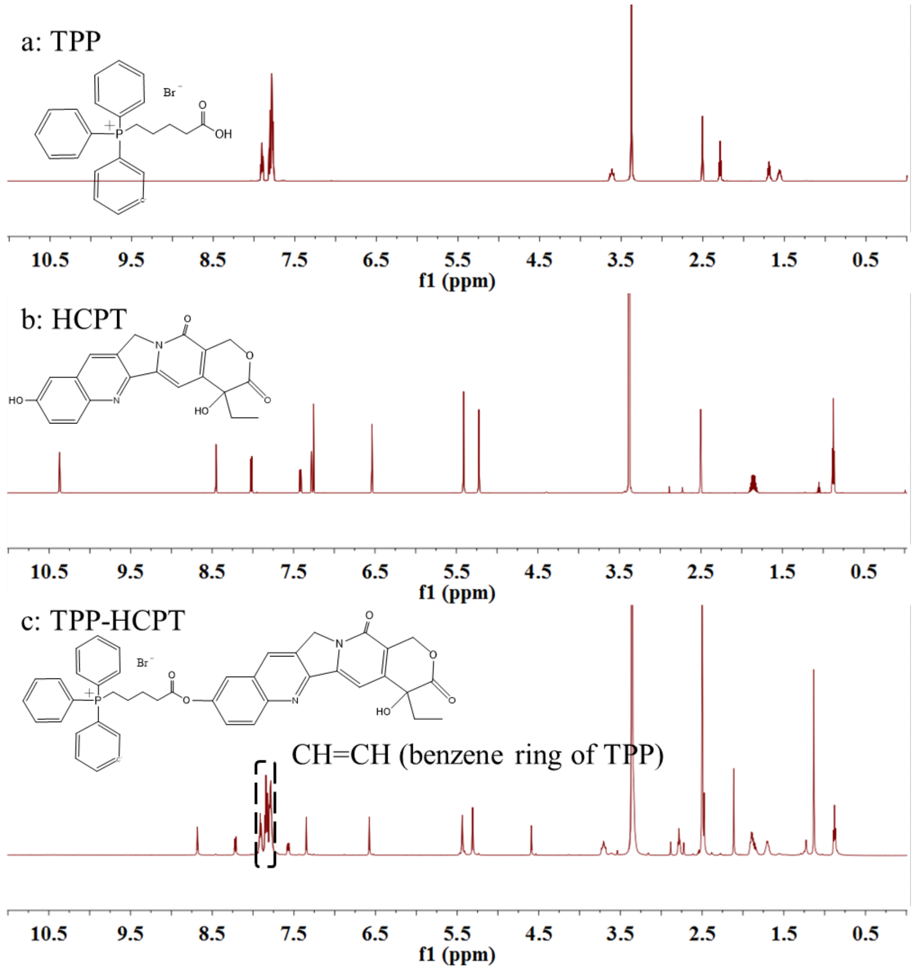

Figure S1. Proton NMR spectra of TPP(a), HCPT(b) and TPP-HCPT conjugate (c).

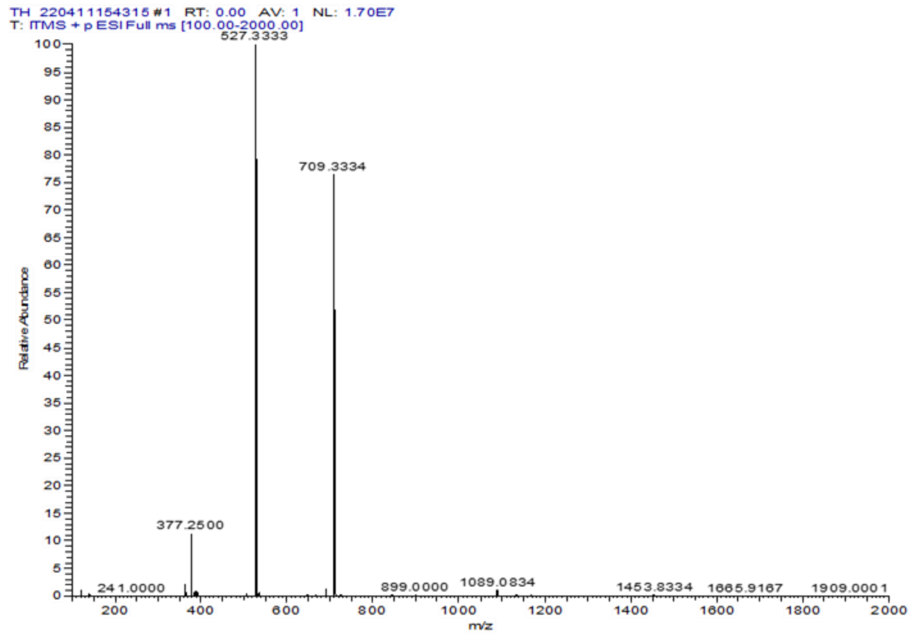

Figure S2. ESI-MS of TPP-HCPT (ESI source: I Spary Voltage: 4.50kV; Tube Lens: 110V; Capillary Temp: 275°C; Capillary Voltage: 35V; Sheath Gas Flow Rate: 5arb).

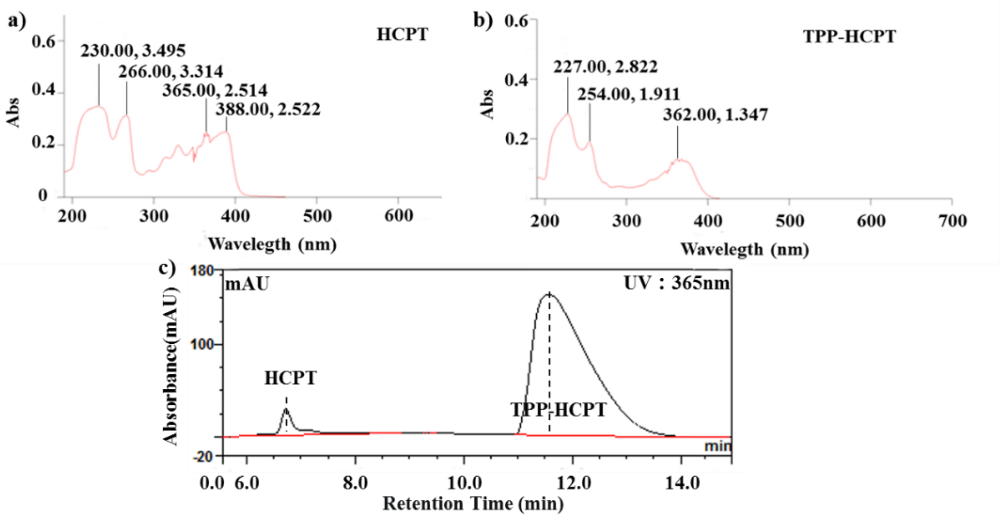

Figure S3. UV full-wavelength scanning of HCPT and TH (190nm-700nm, a and b). High performance liquid chromatography (HPLC) of HCPT and TH under the exact same chromatographic conditions (c).

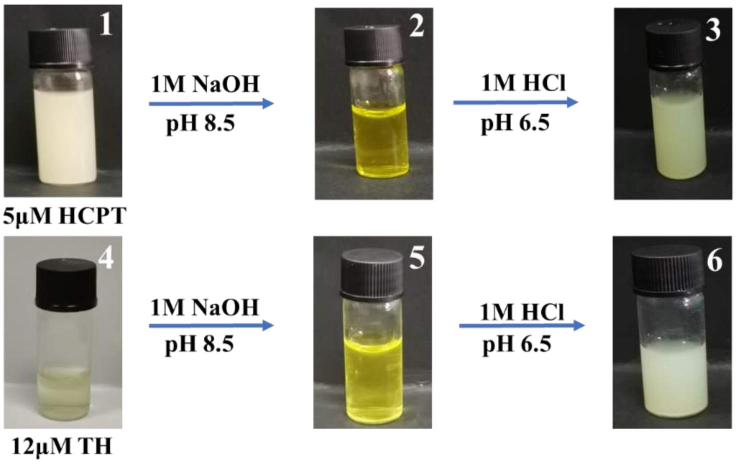

Figure S4. HCPT and TH in deionized water (1 and 4), dissolved in alkaline solution (pH8.5) (2 and 5), and the subsequent aggregation when acidified to pH 6.5 using 1M HCl.

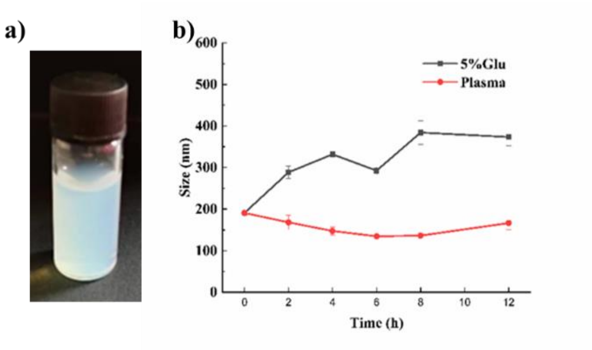

Figure S5. HCPT-loaded TH nanoparticles (a) and their particle size change in 5% glucose and plasma after HA coating (b).

Table S1. Particle size and PDI of TH solution at different concentrations.

Concentration	Size(nm)	PDI	Zeta(mV)
1mg/ml	203.2±12.34	0.312	+0.026
5mg/ml	440.7±32.17	0.264	+1.335
10mg/ml	580.9±13.62	0.330	+3.452

Table S2. Particle size, PDI and zeta potential of HCPT-loaded TH nanoparticles and HA coated HCPT-loaded TH nanoparticles.

Concentration	Size(nm)	PDI	Zeta(mV)
HCPT-loaded TH nanoparticles	155.5±9.96	0.312	+5.62
HA coated HCPT-loaded TH NPs	190.8±6.25	0.208	-3.71

Table S3. The particle size and PDI value of TH nanoparticles (containing 1mg/mL of TH) made from different pharmaceutical adjuvants.

Antisolvent precipitation	Mass ratio of TH:materials	Size (nm)	PDI
P188	1:4	758.5±31.77	0.482
TPGS	2:1	367.9±17.44	0.482
TPGS	1:2	249.3±14.31	0.398
DSPE-PEG_{2000}	4:1	580.9±26.31	0.540
DSPE-PEG_{2000}	1:4	248.5±13.24	0.438
PEG_{5000}-PCL_{4800}	4:1	156.8±10.28	0.418
PEG_{5000}-PCL_{4800}	1:1	134.1±17.28	0.528
PEG_{5000}-PCL_{4800}	1:4	113.4±6.54	0.435
PEG_{3000}-$PLGA_{5000}$	4:1	312.5±6.97	0.311
PEG_{3000}-$PLGA_{5000}$	1:1	234.1±11.25	0.387
PEG_{3000}-$PLGA_{5000}$	1:2	213.1±13.60	0.412
PEG_{3000}-$PLGA_{5000}$	1:3	133.2±8.63	0.352
PEG_{3000}-$PLGA_{5000}$	1:4	86.41±5.93	0.256
chol-PEG_{1k}	4:1	234.7±8.55	0.387
chol-PEG_{1k}	1:4	146.1±6.87	0.347
hyaluronic $acid_{3-10k}$	4:1	422.3±13.14	0.294
hyaluronic $acid_{3-10k}$	1:4	295.3±7.25	0.384
hyaluronic $acid_{40-50k}$	4:1	346.6±18.30	0.786
hyaluronic $acid_{40-50k}$	1:4	292.3±6.91	0.098
